# Association Between Care Utilization and Anxiety Outcomes in an On-Demand Mental Health System: Retrospective Observational Study

**DOI:** 10.2196/24662

**Published:** 2021-01-26

**Authors:** Sarah Kunkle, Manny Yip, Justin Hunt, Watson Ξ, Dana Udall, Patricia Arean, Andrew Nierenberg, John A Naslund

**Affiliations:** 1 Ginger San Francisco, CA United States; 2 Department of Psychiatry and Behavioral Sciences University of Washington Seattle, WA United States; 3 Massachusetts General Hospital Boston, MA United States; 4 Department of Global Health and Social Medicine Harvard Medical School Boston, MA United States

**Keywords:** mental health, digital health, anxiety, telehealth, virtual care, utilization, outcome, retrospective, observational

## Abstract

**Background:**

Anxiety is an extremely prevalent condition, and yet, it has received notably less attention than depression and other mental health conditions from a research, clinical, and public health perspective. The COVID-19 pandemic has only exacerbated growing concerns about the burden of anxiety due to the confluence of physical health risks, economic stressors, social isolation, and general disruption of daily activities.

**Objective:**

This study examines differences in anxiety outcomes by care modality (coaching, teletherapy and telepsychiatry, and combined care) within an on-demand mental health system. We also explore the association between levels of engagement within each care modality and odds of improvement in symptoms of anxiety.

**Methods:**

We conducted a retrospective observational study of individuals who accessed Ginger, an on-demand mental health system. Data were collected from 1611 Ginger members between January 1, 2018, and December 31, 2019. We used logistic regression to assess the association between care modality and improvement in anxiety symptoms. Within each modality, we assessed the association between level of engagement and improvement.

**Results:**

Of 1611 Ginger members, 761 (47.0%) experienced a decrease in anxiety symptoms, as measured by a change from a positive to a negative 2-item Generalized Anxiety Disorder (GAD-2) screen. Among members who still screened positive at follow-up (865/1611, 53%), a total of 192 members (11.9%) experienced a clinically significant score reduction in the full GAD-7 (ie, a score reduction of >5 points), even though their GAD-2 scores were still positive. All modalities showed increased odds of improvement compared to those who were not engaged with coaching or clinical services (“app-only”). Higher GAD-7 intake scores were also associated with decreased odds of improvement.

**Conclusions:**

This study found increased odds of anxiety improvement for all care modalities compared to those who did not engage in care, with larger effect sizes for higher utilization within all care modalities. Additionally, there is a promising observation that those engaged in combined care (teletherapy and text-based coaching) had the greatest odds of anxiety improvement. Future directions include more detailed classifications of utilization patterns and an exploration of explanations and solutions for lower-utilization members.

## Introduction

### Background

Anxiety disorders, including generalized anxiety disorder, panic disorder, and various phobia-related disorders, are prevalent mental health conditions in the United States and globally; large population surveys have shown that about one-third of individuals are affected by an anxiety disorder during their lifetimes [1]. In 2010, anxiety disorders were the sixth leading cause of disability in high-income and low- and middle-income countries [2]. Despite these far-reaching effects, anxiety has received notably less attention than depression from a research, clinical, and public health perspective and often goes unreported or untreated [3,4]. A study by the World Health Organization (WHO) found that only a fifth (20.6%) of participants with an anxiety disorder sought help from health care services, and of those individuals who sought help, 23.2% received no treatment at all [1]. Similarly, in the United States, anxiety disorders are the most common mental health condition, but a large portion of those affected (36.9%) are estimated to go untreated [5].

The COVID-19 pandemic has only exacerbated growing concerns about the burden of anxiety due to the confluence of physical health risks, economic stressors, social isolation, and general disruption of daily activities [6]. A preprint showed that as of April 2020, 1 out of 4 US adults meet the criteria for serious mental distress, 8 times more than a demographically similar sample from 2018 [7]. Data from the Centers for Disease Control and Prevention (CDC) show an increasing percentage of adults experiencing symptoms of anxiety disorder since early April; approximately 35% of adults reported symptoms of anxiety in July 2020 compared to 8.2% in January 2019 to June 2019 [8].

### Treatment

Various treatment options have been shown to be effective for anxiety, including psychotherapy and medication [9]. Psychotherapy techniques include Cognitive Behavioral Therapy (CBT), which teaches people different ways of thinking, behaving, and reacting to anxiety-producing objects and situations. These evidence-based treatments have been effectively delivered as telemedicine offerings, with several systematic reviews indicating that treatment delivered in this manner increases access and reach to care [10-12]. Other benefits of teletherapy include more convenient access, reduced stigma, and greater scalability compared with traditional in-person therapy [13]. Beyond teletherapy, there is also evidence that smartphone interventions can reduce anxiety symptoms [12]. Given the current environment with COVID-19, the ability to reach people in need with virtual care is critical.

Health coaching has also emerged as a potential solution to overcoming traditional shortages of specialist mental health providers. Specific to mental health and anxiety, coaching can work similarly to psychotherapy in addressing symptoms through positive psychology, mindfulness, motivational interviewing, strength, and solution-oriented focuses, among other techniques [14]. A systematic review concluded that health coaching could motivate change in the lifestyle behavior of patients with chronic illness, leading to improvements in both physical and mental health status [15]. Recent studies focused on text-based coaching interventions have shown significant improvements in mental health outcomes equivalent to treatment as usual, namely, in-person and telephone counseling [16,17]. Because there can be significant heterogeneity in these types of interventions, researchers and clinicians have published guidance on developing protocols for text-based coaching in digital mental health interventions [17].

### Study Objectives

There is an established evidence base for anxiety treatments. However, there is a need to understand what is happening “in the wild” versus in controlled settings to understand if members are achieving expected outcomes, to further our understanding of how evidence-based interventions are implemented within new care delivery models, and to potentially uncover new areas for future study [18-20]. Thus, this study examines differences in anxiety outcomes by care modality (coaching, teletherapy and telepsychiatry, and combined care) within an on-demand mental health system. We also explore the association between levels of engagement within each care modality and odds of improvement in symptoms of anxiety. We hypothesize that those engaged in multiple modalities will show greater odds of improvement compared to those engaged in a single care modality, and that within each care modality, more consistent and regular engagement will be associated with greater odds of improvement.

## Methods

### Overview

This is a retrospective observational study of individuals who accessed Ginger, an on-demand mental health system. Data were collected from members of Ginger between January 1, 2018, and December 31, 2019. This time period was chosen because it reflects the approximate timing of when Ginger began to provide care to members via its employer business.

### Participants

Study participants have access to the Ginger system as part of their employer or health plan benefits. Internal clinical protocols include the following exclusionary criteria where self-directed telehealth is likely not appropriate and where more specialized and urgent psychiatric services are required: (1) active suicidal ideation; (2) active high-risk self-harm behavior; (3) 2 or more hospitalizations within the past 6 months, or 1 hospitalization in the past month for psychiatric reasons; (4) certain symptoms of psychosis that are poorly managed (eg, member is not medication compliant or symptoms are unresponsive to treatment) and are likely incompatible with telehealth; (5) a primary diagnosis of a substance use disorder, or moderate-to-severe substance abuse issues, due to the high complexity, severity, and risk frequently associated with such members, as well as the need for specialized care; (6) active eating disorders with symptoms considered to be high risk; (7) ongoing grave disability, including certain patients who are bipolar with active mania/hypomania or mixed episodes who are unmedicated or who have poor compliance with a medication regimen over time; (8) 2 or more medical hospitalizations in the last month, due to the high likelihood that the individual has a poorly controlled medical condition that requires close monitoring.

For this study, we included Ginger users aged 18 years or older who downloaded the app during the study data collection period and screened positive for anxiety on the 2-item Generalized Anxiety Disorder (GAD-2) intake survey.

### Procedures

#### The Ginger System

Ginger provides members with access to behavioral health coaching, teletherapy, telepsychiatry, and self-guided content and assessments, primarily via a mobile app platform. After downloading the mobile app, users are able to start texting with a behavioral health coach within minutes of requesting to connect. Ginger coaches are full-time employees who have an advanced degree in a field related to mental health or have accredited coach certification [21,22]. While many users are solely engaged with text-based coaching services, some will request or require escalation to clinical services (teletherapy or telepsychiatry), depending on preference or clinical severity. Examples of situations that require escalation include individuals with chronic mental illness and severe trauma, the potential to harm oneself or others, and significant mental instability (hallucinations, delusions, extreme mood swings, etc). When members are escalated to therapy or psychiatry, they may continue working with a coach, provided that they also seek additional specialized care concurrently. Coaching can continue supporting them in addressing day-to-day goals and challenges and act as an adjunct to the care plan put in place by their therapist or psychiatrist [23].

#### Data Collection

Ginger uses the 2-item and 7-item Generalized Anxiety Disorder questionnaires (GAD-2 and GAD-7) to assess and track anxiety, both of which have been validated and shown to have good operating characteristics for all anxiety disorders [24]. Additionally, the GAD-2 has been shown to be sensitive to treatment change and, thus, an efficient measure of treatment progress and outcomes [25].

There is no strict guidance on administration protocol; however, an accepted approach is to use the GAD-2 (the first 2 questions of the GAD-7) to screen for anxiety disorders in clinical practice, followed by the remaining 5 items of the full GAD-7 for patients who receive positive results on screening with the first 2 items [3]. Ginger users are prompted to answer the GAD-2 questionnaire through the platform interface when they begin using the Ginger app. Individuals that score a 2 or above for either question are then prompted to complete the full GAD-7. Ginger administers the survey every 2 weeks to users who screen positive and every 3 months for users who screen negative to monitor symptom response and assess if additional care is warranted. Survey completion is not required so as not to withhold support from members who decline to answer the questions but are still interested in accessing the Ginger services.

### Measures

#### Anxiety Symptoms

Symptom improvement is assessed using the GAD-2. For the purpose of this study, we defined *improvement* as individuals who experienced a change from a positive screen to a negative screen at follow-up. A negative screen was defined as a response score for each question of less than 2 (ie, a response of “not at all” or “several days”). A positive screen was defined as a response score for either question of 2 or greater (ie, a response of “more than half the days” or “nearly every day”). Thus, this improvement can be interpreted as an individual’s reduced frequency of reporting key anxiety symptoms (“feeling nervous, anxious, or on edge” or “not being able to stop or control worrying”); more specifically, it reflects a change in experiencing these symptoms for more than half the days or nearly every day to not experiencing them at all or for only several days over the past 2 weeks. As a secondary analysis for users who screened positive at follow-up and completed a full GAD-7 survey, we also looked at a clinically significant reduction in score, defined as a score reduction of >5 points [26-28].

#### Utilization

We calculated utilization based on product user behavior data, including the number of coaching sessions and messages, number of clinical (therapy and psychiatry) appointments completed, and length of time engaged. Prior studies have similarly used these metrics on virtual interactions as utilization measures [29-31].

We categorized utilization levels based on Ginger clinical protocols, external guidelines, and supporting literature. In general, a typical treatment period consists of 8-12 weeks, with weekly interactions to check in on progress. However, there are certain situations in which the duration of treatment is modified (either extended or shortened) based on member-specific circumstances (clinical presentation, covered sessions, the goodness of fit between clinician and member, and life circumstances), which is consistent with recommendations from the literature [17,32]. For example, Ginger clinical protocols state that maintenance and termination can be considered if there is a response by 4-8 weeks. As Ginger members achieve improvements in their GAD scores, they could be moved to a lower level of intervention to ensure therapists are working at the “top of their license” and the system is efficiently using scarce clinical resources. Additionally, some serious and persistent mental illnesses require ongoing and chronic medication management and, thus, a longer treatment duration.

#### Care Modality

Our first set of models assesses the association between care modality and improvement. We considered 5 categories of members: (1) only accessed the Ginger app, (2) interacted with a coach or clinician but did not meet a minimum threshold for engagement, (3) only interacted with a behavioral health coach via text, (4) only interacted with a clinician (therapist or psychiatrist) via video, (5) interacted with both a behavioral health coach and clinician. Table 1 summarizes care modality categorization and rationale.

**Table 1:** Care modality definitions and rationale.

Category	Definition	Rationale
App only	<14 messages sent to coach and 0 clinical sessions	These are members who have not attended a clinical session or completed a full coaching interaction. The 14-message threshold is based on internal analyses of what constitutes a “typical” intake coaching session.
Minimal care utilization	<4 weeks of coaching, or the average days between interactions is ˃14 days, and <2 clinical sessions	These are members who have completed a coaching or clinical session but received minimal therapeutic intervention based on their length of engagement. For coaching, it is not uncommon for an initial consultation to take place over multiple sessions and weeks.
Coaching	≥4 weeks of coaching, and average days between interactions is ≤14 days	Coaches work to get members on a weekly schedule. Prior to 4 weeks of engagement, members are unlikely to achieve a meaningful reduction in symptoms.
Clinical	≥2 clinical visits	The first clinical session is generally considered an “information gathering” intake session. Members generally begin receiving active intervention during their second session.
Combined	≥4 weeks of coaching, and average days between interactions is ≤14 days, and ≥2 clinical sessions	These are members who are engaged with both a coach and clinician, meeting the criteria for both coaching and clinical engagement.

#### Coaching Utilization

In our coaching-only analyses, we considered members who had exchanged at least 14 messages with a coach and created categories based on both the length and the frequency of their interaction (Textbox 1). In general, this categorization allows us to understand associations by months of engagement, from less than a month (“minimal”) to more than 3 months (“high”).

**Categorizations for different care modality utilization.**

**Coaching utilization:**

- Minimal:
  less than 4 weeks, or the average days between interactions is greater than 14 days
- Low:
  4-8 weeks, with average days between interactions <15 days
- Medium:
  9-12 weeks, with average days between interactions <15 days
- High:
  13+ weeks, with average days between interactions <15 days

**Clinical utilization:**

- Minimal:
  1 session
- Low:
  2-6 sessions
- Medium:
  7-12 sessions
- High:
  13+ sessions

**Combined (coaching + clinical) utilization:**

- Minimal:
  total score=0
- Low:
  total score=1-3
- Medium:
  total score=4-5
- High:
  total score=6

#### Clinical Utilization

In our clinical-only analyses, we created 4 categories based on the member’s number of sessions (Textbox 1). We decided on these cutpoints because the first session is generally considered an intake session, with minimal therapeutic intervention, and since many commercial contracts and Employee Assistance Programs (EAPs) cover up to 6 sessions, this categorization allows us to understand differences in outcomes for those who are on either side of this cutpoint. Finally, 12 sessions is generally considered the upper limit for the recommended course of treatment, although sessions may be extended for serious and persistent mental illnesses that require ongoing and chronic medication management.

#### Combined (Coaching + Clinical) Utilization

In our coaching with clinical care (combined) analyses, we considered both a member’s coaching and clinical utilization level to calculate an overall utilization level. Based on the coaching-only and clinical-only model specifications, we assigned the following values to calculate both a coach and clinical score: minimal=0, low=1, medium=2, and high=3. Finally, we calculated a total score (coach score + clinical score) to categorize combined coaching and clinical utilization (Textbox 1).

### Data Management and Analysis

Data for this study were processed using Looker (Looker Data Sciences Inc), a business intelligence and data analytics platform. Data were analyzed in Python and exported to spreadsheets for final analysis and review. We first looked at descriptive statistics of our measures for users who completed GAD questionnaires, segmented by individuals who experienced an improvement versus those who did not. We performed chi-square tests for categorical variables, *t* tests for continuous variables, and Mood median tests for medians to assess differences in characteristics between those who improved versus those who did not.

Given the binary nature of our dependent outcome variable (“improved” vs “did not improve”), we used logistic regression modeling, a common statistical method for quantifying the relationship between various factors and a binary clinical outcome (ie, dependent variable). Our data further meet the assumptions of logistic regression, including independent observations and little or no multicollinearity among the independent variables [33]. Our first set of models assessed the association between care modality and improvement in anxiety symptoms. We assessed the association between level of engagement and improvement within each modality, and we adjusted for baseline anxiety score with the reference group denoted as the “app-only” group (ie, those not engaged with a coach or clinician) as this is the lowest intensity intervention of all options. For each model, we also calculated the Hosmer-Lemeshow goodness-of-fit statistic [34].

### Ethics Statement

This is a secondary analysis of pre-existing de-identified data. The study team does not have access to participant identifying information and does not intend to recontact participants. Ginger’s research protocols and supporting policies have been reviewed and approved by Advarra’s institutional review board in accordance with the US Department of Health and Human Services regulations at 45 CFR 46 [35].

## Results

### Participant Demographics and Characteristics

Based on our inclusion criteria, 4369 individuals were eligible for this study. Of users who screened positive for anxiety, 1611 users (36.9%) completed a follow-up survey at least 14 days after intake and were included in our analysis.

Table 2 shows descriptive characteristics of Ginger platform users, categorized by individuals who experienced a change in GAD screen (ie, from a positive screen to a negative follow-up screen) and those who did not (positive follow-up screen). A total of 1611 individuals initially screened GAD-2 positive for anxiety symptoms, completed a full GAD-7, completed a follow-up screen, and were included in this analysis. Of these 1611 individuals, 756 (46.9%) experienced a decrease in anxiety symptoms as measured by a change from a positive to a negative GAD-2 screen. Among members who still screened positive at follow-up (855/1611, 53.1%), a total of 192 members (11.9%) experienced a clinically significant score reduction in the full GAD-7 (ie, a score reduction of ˃5 points), even though their GAD-2 scores were still positive.

Gender and age data were missing for a large portion of the sample, as this is optional information provided in employer eligibility files. For those users who had reported gender (560/1611, 34.5%), 371 (66.3%) were female and 187 (33.3%) were male. For those users who had available date of birth information (996/1611, 61.8%), 131 (13.2%) were 18-24 years of age, 522 (52.4%) were 25-34 years of age, 220 (22.1%) were 35-44 years of age, 121 (12.1%) were 45-64 years of age, and 2 (0.2%) were 65 years of age or older.

In addition to demographics, Table 2 also reports care modality, levels of utilization, satisfaction scores, and clinical [Patient Health Questionnaire (PHQ) and GAD] intake scores for those who screened negative at follow-up and those who screened positive at follow-up. Compared to those who screened positive at follow-up, those who screened negative at follow-up (ie, “improved”) were significantly less likely to have only engaged with the app or have minimal care utilization. They also tended to have higher levels of utilization within each modality and lower levels of anxiety at intake.

**Table 2:** Characteristics of the study cohort (n=1611).

Characteristic		All participants (N=1611)	Negative follow-up screen (n=756)	Positive follow-up screen (n=855)	*P* value
**Intake year, n (%)**					.49
	2018	581 (36.06)	266 (35.19)	315 (36.84)	
	2019	1030 (63.94)	490 (64.81)	540 (63.16)	
**Gender, n (%)**					.26
	Female	371 (23.03)	174 (23.02)	197 (23.04)	
	Male	187 (11.61)	98 (12.96)	89 (10.41)	
	Other	2 (0.12)	2 (0.26)	0 (0.00)	
	No response	1051 (65.24)	482 (63.76)	569 (66.55)	
**Age in years, n (%)**					.36
	18-24	131 (8.13)	57 (7.54)	74 (8.65)	
	25-34	522 (32.40)	263 (34.79)	259 (30.29)	
	35-44	220 (13.66)	102 (13.49)	118 (13.80)	
	45-64	121 (7.51)	57 (7.54)	64 (7.49)	
	≥65	2 (0.12)	2 (0.26)	0 (0.00)	
	No response	615 (38.18)	275 (36.38)	340 (39.77)	
**Care modality, n (%)**					<.001
	App only	144 (8.94)	50 (6.61)	94 (10.99)	
	Minimal care utilization	625 (38.80)	271 (35.85)	354 (41.40)	
	Coaching only	366 (22.72)	183 (24.21)	183 (21.40)	
	Clinical only	385 (23.90)	197 (26.06)	188 (21.99)	
	Combined (coaching + clinical)	91 (5.65)	55 (7.28)	36 (4.21)	
**Engagement, median (IQR)**					
	Coaching sessions	3 (2-7)	4 (2-8)	3 (2-6)	.01
	Coaching messages	54 (21-127)	62 (26-161)	48 (18-105)	.003
	Clinical appointments	6 (3-12)	7 (3-13)	5 (2-11)	.003
	Therapy appointments	6 (2.5-11)	6 (3-12.75)	5 (2-10)	.06
	Psychiatry appointments	3 (2-5)	2 (1.25-4.75)	3 (2-5)	.30
	Days from intake to last interaction	56 (28-105)	70 (29-126)	43 (16-98)	<.001
**Member satisfaction, mean (SD)**					
	Coach star rating	4.60 (0.68)	4.64 (0.62)	4.55 (0.73)	.04
	Clinical star rating	4.75 (0.66)	4.80 (0.61)	4.70 (0.70)	.14
**GAD^a^ intake, n (%)**					<.001
	0-4: minimal anxiety	4 (0.25)	3 (0.40)	1 (0.12)	
	5-9: mild anxiety	226 (14.03)	134 (17.72)	92 (10.76)	
	10-14: moderate anxiety	705 (43.76)	367 (48.54)	338 (39.53)	
	15-21: severe anxiety	676 (41.96)	252 (33.33)	424 (49.59)	
**PHQ^b^ intake, n (%)**					<.001
	Negative screen	680 (42.21)	348 (46.03)	332 (38.83)	
	0-4 minimal or none	3 (0.19)	2 (0.26)	1 (0.12)	
	5-9 mild	58 (3.60)	35 (4.63)	23 (2.69)	
	10-14 moderate	305 (18.93)	155 (20.50)	150 (17.54)	
	15-19 moderately severe	333 (20.67)	133 (17.59)	200 (23.39)	
	20-27 severe	232 (14.40)	83 (10.98)	149 (17.43)	

^a^GAD: Generalized Anxiety Disorder.

^b^PHQ: Patient Health Questionnaire.

Table 3 reports the results of our primary model examining the association between care modality and anxiety symptom improvements. All modalities (coaching, clinical, combined) showed increased odds of improvement compared to those who were not engaged with coaching or clinical services (“app only”). A higher GAD-7 intake score was also associated with decreased odds of improvement.

**Table 3:** Associations between care modality and improvement (n=1611).

Modality	Model 1	
	Odds ratio^a^	95% CI
App only	N/A^b^	N/A
Minimal care utilization	1.45	0.98-2.12
Coaching only	1.90	1.27-2.86
Clinical	1.97	1.32-2.96
Combined	3.26	1.87-5.68
GAD-7 intake score	0.90	0.88-0.92

^a^Odds ratio obtained by exponentiation of the regression coefficients.

^b^N/A: not applicable.

A Hosmer-Lemeshow test failed to reject the null hypothesis, indicating goodness of fit, X^2^ (8, N=1611)=8.9, P=.35.

**Figure 1:**
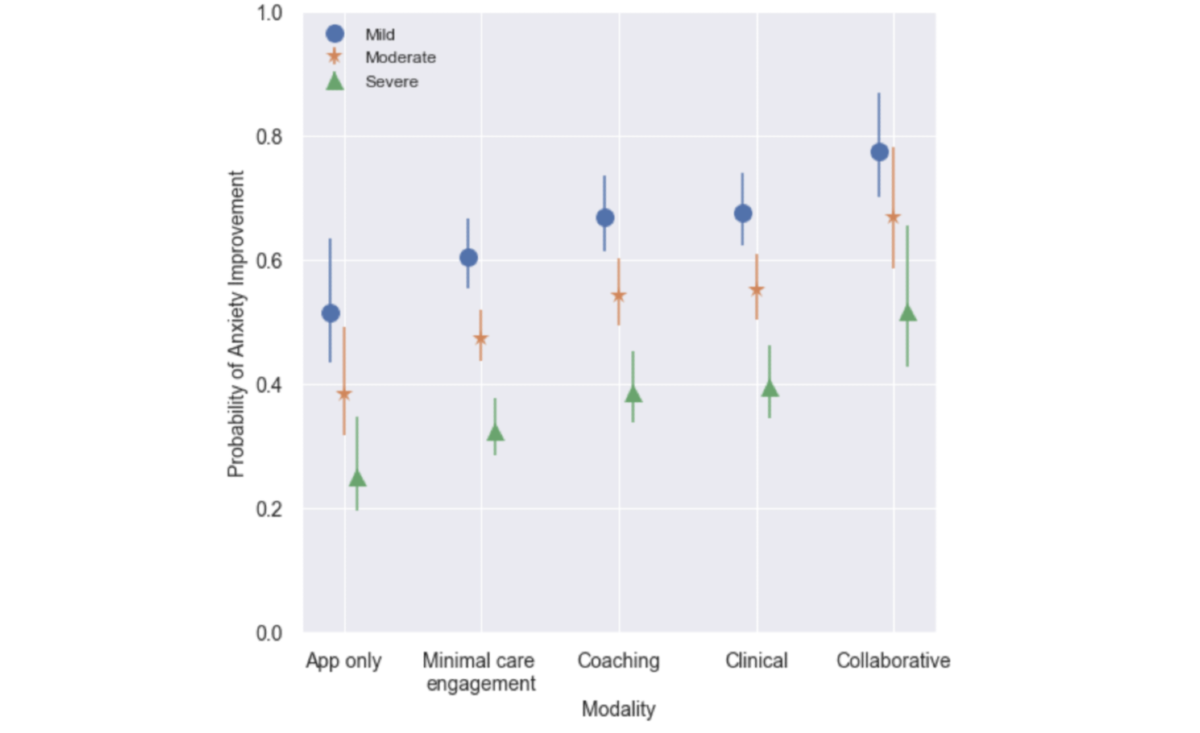
Probability of anxiety improvement by care modality and level of intake severity. Shapes represent the expected mean probability of improvement by modality and intake severity; lines represent the corresponding 95% confidence interval.

These results are shown graphically as probability of anxiety improvement by care modality and levels of intake severity in Figure 1. For all figures, shapes represent the expected mean probability of improvement by modality and intake severity; lines represent the corresponding 95% confidence interval.

### Coaching-Only Cohort

Table 4 reports outputs for the model examining the association between utilization and anxiety symptom improvement for the text-based coaching-only cohort. Compared to the minimal-utilization reference group (those who sent less than 14 messages to a coach, engaged less than 4 weeks, or the average days between interactions were greater than 14 days), only the high-utilization group had significantly increased odds of improvement. This association remained after adjustment for baseline severity (GAD-7 intake score).

A Hosmer-Lemeshow test failed to reject the null hypothesis, indicating goodness of fit, X^2^ (8, N=1080)=9.14, P=.33.

**Figure 2:**
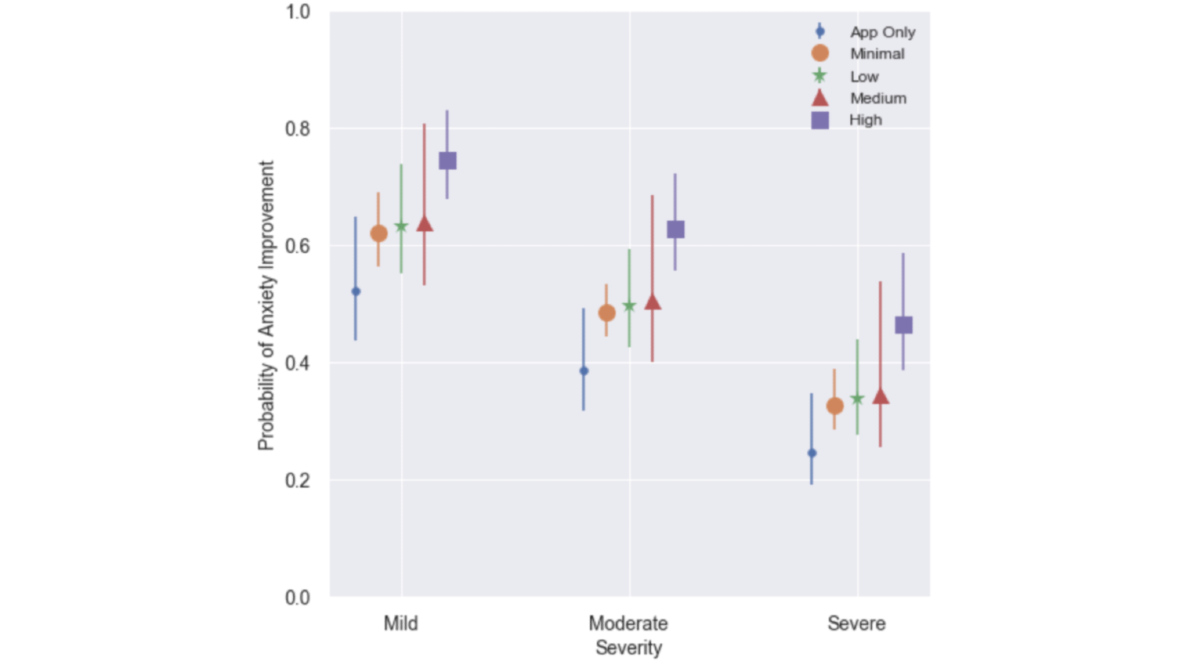
Probability of anxiety improvement by utilization and intake severity among the text-based coaching-only cohort (n=936). Shapes represent the expected mean probability of improvement by modality and intake severity; lines represent the corresponding 95% confidence interval.

These results are shown graphically as probability of anxiety improvement by coaching utilization and intake severity in Figure 2.

**Table 4:** Associations between utilization and improvement among the text-based coaching-only cohort (n=936).

Modality	Model 2	
	Odds ratio^a^	95% CI
App Only	N/A^b^	N/A
Minimal	1.50	1.02-2.21
Low	1.57	0.98-2.52
Medium	1.63	0.87-3.08
High	2.70	1.65-4.41
GAD-7 intake score	0.89	0.87-0.92

^a^Odds ratio obtained by exponentiation of the regression coefficients.

^b^N/A: not applicable.

### Clinical-Only Cohort

Table 5 reports outputs for the model examining the association between utilization and anxiety symptom improvement for the clinical-only cohort. Compared to the minimal-utilization reference group (those who only attended 1 therapy session), those in the low, medium, and high utilization categories had increased odds of improvement, with odds ratios increasing ordinally with each category. After adjusting for GAD-7 intake score, only the high utilization coefficient remained significant.

**Table 5:** Associations between clinical utilization and improvement among the clinical-only cohort (n=205).

Modality	Model 3	
	Odds ratio^a^	95% CI
App Only	N/A^b^	N/A
Minimal (1 session)	0.75	0.29-1.92
Low (2-6 sessions)	1.91	1.13-3.21
Medium (7-12 sessions)	2.24	1.04-4.83
High (≥13 sessions)	2.30	1.07-4.93
GAD-7 intake score	0.90	0.85-0.96

^a^Odds ratio obtained by exponentiation of the regression coefficients.

^b^N/A: not applicable.

A Hosmer-Lemeshow test failed to reject the null hypothesis, indicating goodness of fit, X^2^ (8, N=349)=6.44, P=.50.

**Figure 3:**
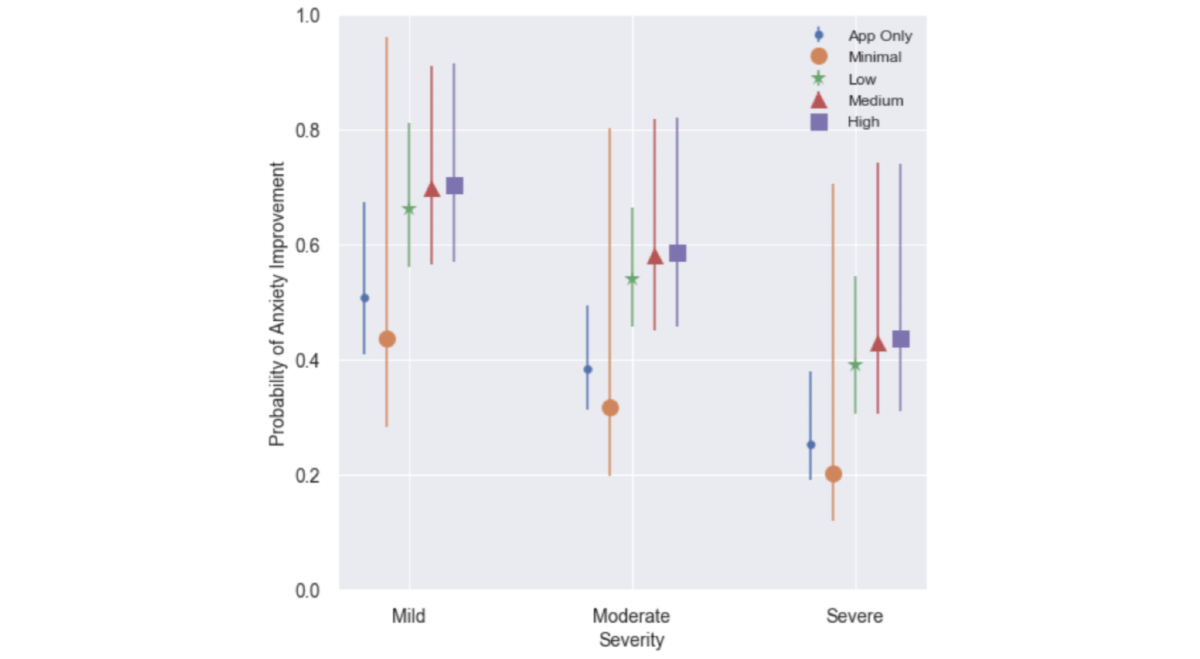
Probability of anxiety improvement by clinical utilization and intake severity among the clinical-only cohort (n=205). Shapes represent the expected mean probability of improvement by modality and intake severity; lines represent the corresponding 95% confidence interval.

These results are shown graphically as probability of anxiety improvement by clinical utilization and intake severity in Figure 3.

### Combined (Coaching and Clinical Services) Cohort

Finally, Table 6 reports outputs for the model examining the association between utilization and anxiety symptom improvement for individuals engaged in both coaching and clinical services. Compared to the low-utilization group, there were significantly increased odds of improvement for the high utilization group but not for the low- and medium-utilization group.

A Hosmer-Lemeshow test failed to reject the null hypothesis, indicating goodness of fit, X^2^ (8, N=470)=7.11, P=.53.

**Figure 4:**
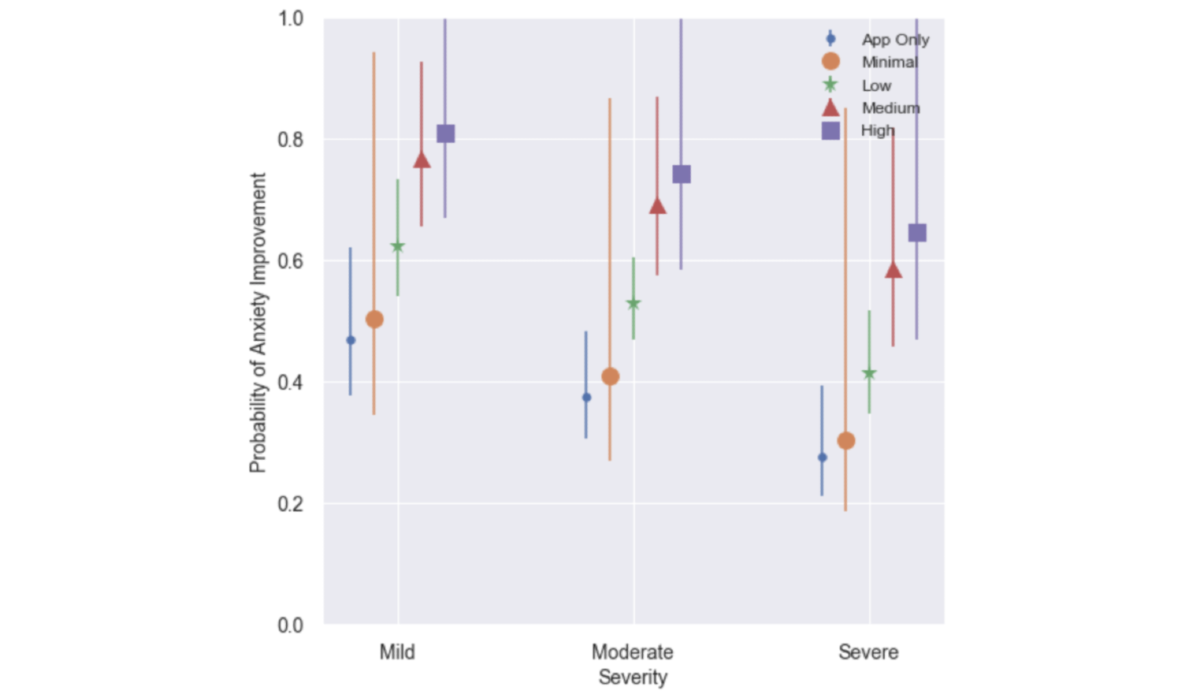
Probability of anxiety improvement by utilization and intake severity among the combined-care cohort (n=326). Shapes represent the expected mean probability of improvement by modality and intake severity; lines represent the corresponding 95% confidence interval.

These results are shown graphically as probability of anxiety improvement by coaching and clinical utilization and intake severity in Figure 4.

**Table 6:** Associations between utilization and improvement among the combined-care cohort (n=326).

Modality	Model 4	
	Odds ratio^a^	95% CI
App Only	N/A^b^	N/A
Minimal	1.15	0.44-2.99
Low	1.87	1.22-2.87
Medium	3.76	1.79-7.89
High	4.85	1.6-14.7
GAD-7 intake score	0.93	0.88-0.97

^a^Odds ratio obtained by exponentiation of the regression coefficients.

^b^N/A: not applicable.

## Discussion

### Principal Findings

In this study, we examined differences in anxiety outcomes by care modality—coaching, clinical (teletherapy and telepsychiatry), and combined (coaching and clinical) care—within an on-demand mental health system, as well as the association between levels of utilization within each care modality and improvement in anxiety symptoms. Our primary model examining the association between improvement and care modality found increased odds of improvement for all care modalities compared to the reference group (those who did not engage in any coaching or clinical services). This aligns with existing literature that finds that most forms of treatment are better than nothing, further highlighting the need to get even low-intensity treatments to individuals who need help. Our outcomes are also in line with prior observational research estimating GAD recovery rates of 30%-60%, depending on treatment and individual characteristics [36,37].

We also found the largest effect size for the combined-care (coaching and clinical) group, suggesting that engaging in multiple levels of care might be more effective for treating anxiety. More specifically, coaching might provide an added benefit of longitudinal support toward goals between episodic clinical visits. This is a novel finding given the limited research focused on text-based coaching and this form of combined care. It also suggests that more intensive forms of digital mental health services appear to contribute to greater improvement in outcomes, which is important for considering the scalability of these programs. It is important to note that a relatively small percentage of our study cohort was engaged in combined care, suggesting a need to promote this modality more broadly.

Our data suggest that while all treatment modalities appeared to offer comparable benefits in managing anxiety, the largest effects were observed among those who engaged in services for at least 13 weeks of care. This mirrors data from clinical trials of in-person care for anxiety, where the largest effects are for those who receive more frequent sessions. This is also consistent with most evidence-based protocols of 8-12 sessions, as these sessions are not always completed at a weekly cadence [17].

Strengths of this study include the real-world setting and relatively large sample size, which allow us to observe varying levels of digital mental health support for anxiety among individuals seeking care for their mental concerns. While limited in several ways, this design has an important benefit in not being constrained by the strict requirements for controlled clinical trials. Additionally, due to the virtual nature of the system, we have detailed data on coaching utilization (eg, messaging volume and frequency) that likely would not exist for in-person care settings, and the ability to compare multiple modalities of care. This study is also novel in its ability to analyze data for people engaged in multiple modalities of care (ie, teletherapy and text-based coaching).

### Limitations

There are several limitations to this study. As our dataset is limited to people who completed surveys, our results are not necessarily generalizable to all members (ie, those who drop off or do not complete surveys). Furthermore, our results cannot generalize to individuals who do not have access to this system. We also had a relatively large amount of missing data for gender and age, which limited our ability to stratify analyses by key demographics. Due to the survey design of this system and efforts to maintain anonymity and protect the privacy of members, we were unable to study race/ethnicity, socioeconomic status, living situation, history of trauma or other mental illnesses, and other factors that could affect treatment response. However, because the Ginger platform is offered through employers, we know that the survey respondents are working-age adults, suggesting that these findings may generalize to the professional workforce and those enrolled in health benefits through their employer.

Another potential limitation is that we had to rely on GAD-2 rather than GAD-7 to assess anxiety symptom improvement. This is due to the survey system design, which aims to avoid response burnout among users. This limits our ability to assess certain anxiety symptoms; however, GAD-2 represents 2 core anxiety symptoms and has been shown to have good sensitivity and specificity in the diagnosis of the most common anxiety disorders [4].

It is also important to note that we are likely underestimating the number of individuals who experienced a clinically meaningful improvement in their anxiety symptoms, as some members screened positive at follow-up (and those were not classified as “improved” in our models) but experienced a reduction in GAD-7 score. If we include the commonly accepted definition of a 5-point reduction in score, an additional 11.9% (192/1617) of the cohort would be classified as “improved.” Finally, we lack a control group to understand what would have happened in the absence of Ginger and to attribute causality, although we are able to understand these associations relative to defined reference groups.

This study segues into many directions of future research. We can build upon our understanding of these associations by increasing the collection of demographic data to enhance our understanding of member utilization patterns and user personas, and by adding content analysis of coaching messages. For example, we might consider looking more specifically and in greater depth at the frequency and intensity of coaching utilization and different patterns of coaching and clinical utilization (sequential vs concurrent). Additionally, we plan to explore how the different treatment modalities contribute to improved outcomes, digging into the “mechanism of action” to better understand how to replicate aspects of the platform that work well to support the larger rollout of these services to users.

### Conclusion

This study found increased odds of anxiety improvement for all care modalities compared to those who did not engage in care, with larger effect sizes for higher utilization within all care modalities. Additionally, there is a promising observation that those engaged in combined care (teletherapy and text-based coaching) have the greatest odds of anxiety improvement. Future directions include more detailed classifications of utilization patterns and exploring explanations and solutions for lower utilization members.

## Abbreviations

GAD: Generalized Anxiety Disorder
PHQ: Patient Health Questionnaire
